# Sustainable Management Methods of Orchard Insect Pests

**DOI:** 10.3390/insects12010080

**Published:** 2021-01-18

**Authors:** Estrella M. Hernández-Suárez, Francisco Beitia

**Affiliations:** 1 Instituto Canario de Investigaciones Agrarias (ICIA), P.O. Box 60, La Laguna, 38200 Santa Cruz de Tenerife, Canary Islands, Spain; ehernand@icia.es; 2 Unit of Entomology, Plant Protection and Biotechnology Center, Instituto Valenciano de Investigaciones Agrarias (IVIA), Ctra CV-315, Km 10′7, 46113 Moncada, Valencia, Spain

## Introduction

The current need for sustainable resource management is increasingly urgent, as demand for agricultural commodities is rising rapidly as the world’s population grows. However, agricultural intensification is one of the main drivers of the global biodiversity crisis and global decline in insects [1,2]. Additionally, an impressive amount of research has been published regarding the many detrimental effects of pesticides, which had been the main approach developed for pest control in the first half of the previous century, but which has been progressively replaced by the use of biopesticides and other sustainable methods (biological, physical and other non-chemical methods) to provide satisfactory pest control and implementing Integrated Pest Management (IPM) [3,4,5].

The goal of current sustainable agriculture is to provide society’s food in the present, in the today’s changing environmental scenario, without compromising ability of future generations to get their own needs, and all this by integrating three main objectives into their work: a healthy environment, economic profitability, and social and economic equity. In this context, the IPM in a sustainable agriculture requires progressively replacing use of non-renewable and unsustainable inputs while enhancing the application of eco-friendly practices balancing the double objectives of efficient plant protection and reducing environmental risk.

Different approaches have received attention for controlling orchard pests in a way that results in the reduction in the overall amount of insecticide applied in this agro system and moves towards a more sustainable agriculture. Among all these approaches, the following can be included:

✽ Cultural control practices, like the intercropping, which is the simultaneous cultivation of plant species in same orchard for a considerable proportion of their growing periods [6] to increase overall crop productivity and profitability, improve pest and disease management, and gain better efficiency in the use of nutrient, water, and light resources [7].
✽ Induced plant resistance: A defense system within plants which allows them to resist attacks from pests such as fungal or bacterial pathogens or insects. The defense system reacts to the external attack with physiological changes, triggered by the generation of proteins and chemicals that lead to activation of the plant’s immune system [8].
✽ Soil steaming as disinfection of soil-borne insect pests: This technique is regaining popularity to control insect pests in agriculture in recent years. It is an effective sustainable alternative to chemicals and fumigants to disinfect soil in open field, in which hot steam heats up the substrate to temperatures that kill or inactivate soil insects or development phases of some insects [9].
✽ Use of plant extracts as biopesticides: The well-known biocide effect of such extracts converts them in an alternative good source to chemical pesticides due to their safe, eco-friendly and more compatible properties [10].
✽ Semiochemicals for controlling insect pests: These compounds are informative molecules mainly used in plant-insect or insect-insect interactions as alternative or complementary components to insecticide approaches in different IPM strategies. They can be used in various ways such as monitoring, mass trapping, attract-and-kill, push–pull and disruption strategies [11].
✽ Biological control: Where possible, biological and ecological means should be used, promoting naturally occurring biological agents and other inherent strengths as components of total agricultural ecosystems [12].

All the abovementioned approaches, as well as others, related to the sustainability of pest management in orchards will be considered in this Special Issue wherein the design and assessment of ecologically sound strategies should represent the present and the future of the “sustainable management methods of orchard insect pests”.
